# Estimulação do Sistema de Condução Versus Estimulação Biventricular na Insuficiência Cardíaca Crônica: Protocolo para a Análise Econômica do Estudo PhysioSync-HF

**DOI:** 10.36660/abc.20250254

**Published:** 2026-01-26

**Authors:** Sérgio R. R. Decker, Ana Paula Beck da S Etges, André Zimerman, Fernanda D. Alves, Caique M. Ternes, Juliana S. Santos, Leandro Zimerman, Luis Eduardo Rohde, Alexander Dal Forno, André d’Avila, Dhruv S. Kazi, Eduardo G. Bertoldi, Carisi A. Polanczyk

**Affiliations:** 1 Serviço de Clínica Médica Hospital Moinhos de Vento Porto Alegre RS Brasil Serviço de Clínica Médica do Hospital Moinhos de Vento, Porto Alegre, RS – Brasil; 2 Smith Center for Outcomes Research Beth Israel Deaconess Medical Center Harvard Medical School Boston EUA Smith Center for Outcomes Research, Beth Israel Deaconess Medical Center and Harvard Medical School, Boston – EUA; 3 Programa de Pós-Graduação em Cardiologia e Ciências Cardiovasculares Universidade Federal do Rio Grande do Sul Porto Alegre RS Brasil Programa de Pós-Graduação em Cardiologia e Ciências Cardiovasculares, Universidade Federal do Rio Grande do Sul, Porto Alegre, RS – Brasil; 4 MOVE Academic Research Organization Hospital Moinhos de Vento Porto Alegre RS Brasil MOVE Academic Research Organization, Hospital Moinhos de Vento, Porto Alegre, RS – Brasil; 5 Programa de Pós-Graduação em Epidemiologia Universidade Federal do Rio Grande do Sul Porto Alegre RS Brasil Programa de Pós-Graduação em Epidemiologia, Universidade Federal do Rio Grande do Sul, Porto Alegre, RS – Brasil; 6 Hospital Moinhos de Vento Porto Alegre RS Brasil Projetos de Pesquisa, Hospital Moinhos de Vento, Porto Alegre, RS – Brasil; 7 Serviço de Cardiologia Hospital Moinhos de Vento Porto Alegre RS Brasil Serviço de Cardiologia, Hospital Moinhos de Vento, Porto Alegre, RS – Brasil; 8 Hospital SOS Cardio Florianópolis SC Brasil Hospital SOS Cardio, Florianópolis, SC – Brasil; 9 Cardiac Arrhythmia Service Beth Israel Deaconess Medical Center The Harvard Thorndike Electrophysiology Institute Boston EUA Cardiac Arrhythmia Service, Beth Israel Deaconess Medical Center and The Harvard Thorndike Electrophysiology Institute, Boston – EUA; 10 Faculdade de Medicina Universidade Federal de Pelotas Pelotas RS Brasil Faculdade de Medicina, Universidade Federal de Pelotas, Pelotas, RS – Brasil; 11 Hospital de Clínicas de Porto Alegre Porto Alegre RS Brasil Hospital de Clínicas de Porto Alegre, Porto Alegre, RS – Brasil; 12 Instituto para Avaliação e Translação em Saúde para doenças Crônicas e negligenciadas de Alta Relevância Porto Alegre RS Brasil Instituto para Avaliação e Translação em Saúde para doenças Crônicas e negligenciadas de Alta Relevância, IATS-CARE, Porto Alegre, RS – Brasil

**Keywords:** Terapia de Ressincronização Cardíaca, Insuficiência Cardíaca Sistólica, Custos de Cuidados de Saúde

## Abstract

**Fundamento:**

A estimulação do sistema de condução (ESC) surgiu como uma alternativa à estimulação biventricular (EBV) para terapia de ressincronização cardíaca (TRC), com potenciais benefícios clínicos e menores custos. O PhysioSync-HF é um estudo multicêntrico e randomizado que compara essas estratégias sob as perspectivas clínica e econômica em pacientes com insuficiência cardíaca e fração de ejeção reduzida (ICFER).

**Objetivo:**

Descrever a justificativa e o desenho da avaliação econômica baseada em ensaio clínico incorporada ao estudo PhysioSync-HF.

**Métodos:**

O estudo PhysioSync-HF incluiu 179 pacientes com seguimento de 1 ano. Os dados de custo do procedimento serão coletados utilizando a abordagem de custeio baseado em atividades e tempo. Os custos associados ao dispositivo, eventos clínicos adversos e cuidados ambulatoriais durante o seguimento serão estimados por meio de métodos de contabilidade baseados em recursos consumidos. Métodos apropriados serão empregados para tratar dados ausentes, e as análises estatísticas considerarão a distribuição assimétrica das variáveis de custo.

**Resultados:**

O desfecho econômico primário é a diferença entre os grupos no custo médico direto total por paciente ao longo de 1 ano de seguimento (ESC vs. EBV). Os desfechos secundários incluem a decomposição dos custos diretos em componentes e uma análise de impacto orçamentário que estima o efeito anual no sistema de saúde brasileiro se todos os pacientes elegíveis recebessem ESC em vez de EBV.

**Conclusão:**

Aproveitando um ensaio cardiovascular multicêntrico para mensurar os custos da ESC em comparação à EBV, esta avaliação econômica busca identificar oportunidades de redução de custos que possam ampliar o acesso com equidade à TRC para indivíduos com ICFER no Brasil, além de fornecer informações relevantes para outros sistemas de saúde no mundo.

**Registro do Ensaio:**

NCT05572736.

## Graphical abstract

**Figura Central:**
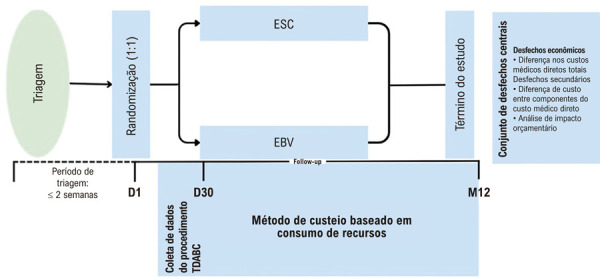
Estimulação do Sistema de Condução Versus Estimulação Biventricular na Insuficiência Cardíaca Crônica: Protocolo para a Análise Econômica do Estudo PhysioSync-HF

Desenho do ensaio com coleta planejada de dados clínicos e econômicos. Dados do TDABC são coletados de D1 até D30; o seguimento do estudo continua até M12. D1: dia 1; D30: dia 30; EBV: estimulação biventricular; ESC: estimulação do sistema de condução; FEVE: fração de ejeção do ventrículo esquerdo; ICFER: insuficiência cardíaca com fração de ejeção reduzida; M12: mês 12; TDABC método de custeio baseado em atividades e tempo (time-driven activity-based costing).

## Introdução

A insuficiência cardíaca com fração de ejeção reduzida (ICFER) é uma condição crônica que afeta cerca de 1,3% da população mundial em todas as faixas etárias, com estimativas mais altas de 3,4% entre adultos e 8,3% entre idosos.^1^ Devido ao envelhecimento populacional, o número de indivíduos afetados continua a aumentar.^2^ Atualmente, cerca de 600 a cada 100.000 pessoas vivem com ICFER no Sul da América Latina, com quase o dobro dessa prevalência na América do Norte, contribuindo para uma carga clínica e econômica substancial e crescente.^1 - 4^

Dentro do grupo de pacientes com ICFER, aqueles com anormalidades de condução representam um subgrupo de maior risco, para o qual terapias individualizadas oferecem benefícios importantes.^5^ A terapia de ressincronização cardíaca (TRC) reduz a morbimortalidade em pacientes com ICFER e bloqueio completo de ramo esquerdo (BRE).^5 - 8^ A estimulação biventricular (EBV) é a técnica padrão para fornecer estimulação sincronizada, enquanto a estimulação do sistema de condução (ESC), que estimula diretamente o sistema de condução intrínseco, surgiu recentemente como uma alternativa promissora.^9^

Do ponto de vista econômico, a ESC pode reduzir custos procedimentais por exigir menos insumos. Essa consideração é particularmente relevante em países de baixa e média renda (PBMR), como o Brasil, onde cerca de 200.000 indivíduos são elegíveis para TRC, mas apenas uma pequena fração a recebe.^3 , 10 , 11^ Os altos custos e a complexidade do procedimento têm limitado a implementação global da TRC e contribuem para a desigualdade no acesso entre pessoas com ICFER.^12 - 14^

A incorporação da coleta de dados econômicos em ensaios clínicos pode alinhar a adoção de novas tecnologias pelos órgãos regulatórios ao seu valor para a sociedade.^15 - 17^ Diante da carga clínica e econômica crescente impulsionada por mudanças demográficas e pelo aumento das doenças crônicas não transmissíveis, lideradas pelas doenças cardiovasculares,^3 , 18 , 19^ essa abordagem permite uma quantificação mais precisa dos custos por meio de métodos de microcusteio e pode catalisar melhorias mais amplas na gestão da saúde.^15 , 16 , 20 - 22^

Entretanto, as avaliações de custo baseadas em ensaios clínicos enfrentam desafios, incluindo heterogeneidade na coleta e análise de dados, assim como a limitada viabilidade de medições detalhadas, o que pode resultar em altas taxas de dados ausentes.^23 - 26^ Essas questões epidemiológicas e metodológicas reforçam a necessidade de descrições metodológicas claras e publicações de protocolos para avaliações econômicas baseadas em ensaios clínicos.

Este manuscrito tem como objetivo descrever a avaliação econômica incorporada ao estudo PhysioSync-HF (NCT05572736), que compara ESC e EBV em pacientes com ICFER. Nossa hipótese central é que a ESC apresentará economia de custos em relação à EBV sob a perspectiva do sistema de saúde e poderá reduzir barreiras financeiras ao encaminhamento e ao acesso à TRC.

## Métodos

### Desenho do estudo

O PhysioSync-HF é um ensaio multicêntrico, randomizado e de não inferioridade que compara a ESC com a EBV em pacientes com ICFER e BRE. Um desfecho secundário chave é a diferença entre os grupos no custo médico direto total por paciente, avaliado sob a perspectiva do sistema de saúde brasileiro ( Tabela 1 ), abrangendo tanto o setor público, o Sistema Único de Saúde (SUS), quanto o setor privado.

**Tabela 1:** – Perspectiva da avaliação de custos incluindo custos médicos diretos totais

Componente de custo	Elementos incluídos	Perspectiva	
		Sistema de Saúde	Sociedade
Custos diretos de saúde pagos por terceiros	Medicamentos/dispositivos; serviços hospitalares; serviços médicos; exames	x	x
Custos diretos de saúde pagos diretamente pelos pacientes	Medicamentos/dispositivos; serviços hospitalares; serviços médicos; exames	x	x
Custos diretos não médicos	Tempo do paciente; tempo de cuidadores não remunerados; transporte		x
Custos indiretos	Perda de produtividade; custo de mortalidade prematura		x
Outros custos indiretos ou intangíveis	Jurídicos; ambientais; outros		x

Destacam-se os custos avaliados nesta análise econômica baseada em ensaio clínico, que são categorizados como despesas médicas diretas.

Os custos incluem aqueles relacionados ao procedimento de TRC e ao acompanhamento subsequente. A Figura Central apresenta o esquema do estudo e o plano de coleta de dados da avaliação econômica incorporada. Os métodos são relatados de acordo com as recomendações do *Standard Protocol Items: Recommendations for Interventional Trials* e, quando aplicável às análises econômicas, com elementos do *Consolidated Health Economic Evaluation Reporting Standards* .^27 , 28^ O protocolo completo do estudo está registrado em ClinicalTrials.gov (NCT05572736).

Um total de 179 pacientes foi incluído entre novembro de 2022 e dezembro de 2023. A coleta e a análise dos dados econômicos abrangerão o seguimento de 12 meses. Os investigadores principais, coordenadores e diretores de pesquisa permanecerão cegos aos resultados econômicos até o travamento do banco de dados. O estudo é financiado pelo Ministério da Saúde do Brasil por meio do Programa de Apoio ao Desenvolvimento Institucional do Sistema Único de Saúde (Proadi-SUS).

### Locais do estudo e centros participantes

O recrutamento ocorreu em ambientes ambulatoriais e hospitalares distribuídos em 14 centros localizados em 11 estados brasileiros (Figura S1). Todos os centros participantes receberam treinamento padronizado para a coleta de dados econômicos.

### População do estudo

Os participantes elegíveis são adultos (≥ 18 anos) com ICFER, fração de ejeção do ventrículo esquerdo (FEVE) ≤ 35%, sintomas de classe funcional II-III da New York Heart Association (NYHA) e BRE com duração do QRS ≥ 130 ms. Todos os participantes deviam estar em terapia medicamentosa otimizada conforme diretrizes e possuir encaminhamento para TRC.

### Intervenção do estudo

Os participantes foram randomizados na proporção 1:1 para ESC ou EBV. A estratégia de ESC inclui estimulação da área do BRE, estimulação do feixe de His ou estimulação septal profunda; caso a ressincronização elétrica permanecesse incompleta, foi permitida a TRC otimizada para o feixe de ramo esquerdo ( *left bundle branch–optimized CRT* , LOT-CRT) com a adição de um eletrodo no seio coronário. O procedimento de EBV seguiu as práticas contemporâneas baseadas em diretrizes e foi realizado pela equipe de eletrofisiologia. O cruzamento entre grupos foi permitido caso a estratégia atribuída se mostrasse malsucedida.

### Desfechos

#### Desfechos clínicos

O desfecho clínico primário do PhysioSync-HF é um composto hierárquico de mortalidade por todas as causas, hospitalização por ICFER, visita de urgência por ICFER e variação da FEVE do basal para 12 meses. A não inferioridade da ESC em relação à EBV será testada inicialmente, com margem definida como razão de chances < 1,20. Caso a não inferioridade seja demonstrada, a superioridade será testada sequencialmente para o mesmo desfecho.

#### Desfecho econômico primário

O desfecho econômico primário (também desfecho secundário chave do ensaio) é a diferença no custo médico direto total por paciente entre os grupos de tratamento. Os custos médicos diretos totais englobam os componentes listados na Tabela 2 . As análises utilizarão uma população modificada por intenção de tratar aos 12 meses, incluindo todos os pacientes randomizados que realizaram o procedimento inicial, de modo a capturar os custos relacionados a cruzamentos precoces ou tardios após a randomização.

**Tabela 2:** – Recursos e estimativas de custo mensurados durante o estudo PhysioSync-HF

Recurso	Fonte da estimativa de custo (caso base)	Potenciais análises de sensibilidade
Hospitalização inicial		
**Dispositivo de TRC**	Perspectiva de compra no mercado	Perspectiva de compra pública vs. privada
**Utilização de mão de obra** ^*^	TDABC	—
**Utilização de instalações hospitalares** ^**†**^	TDABC	—
**Medicamentos**	Registros hospitalares	—
**Exames**	Registros hospitalares	—
**Outros insumos**	Registros hospitalares	—
Seguimento após a alta		
Cuidados ambulatoriais para ICFER	Ghisleni et al. ^36^	—
Visitas urgentes por descompensação de ICFER	CBHPM ^45^	SIGTAP ^35^
Hospitalização por descompensação de ICFER	CBHPM ^45^	SIGTAP ^35^

*A utilização de mão de obra inclui custos de profissionais médicos e não médicos. ^†^A utilização de instalações inclui custos de sala de recuperação anestésica, laboratório de eletrofisiologia, enfermaria e unidade de terapia intensiva. CBHPM: Classificação Brasileira Hierarquizada de Procedimentos Médicos (referência de custos em saúde); ICFER: insuficiência cardíaca com fração de ejeção reduzida; SIGTAP: Sistema de Gerenciamento da Tabela de Procedimentos, Medicamentos e OPM do SUS (referência do setor público); TDABC: time-driven activity-based costing (custeio baseado em atividades e tempo); TRC: terapia de ressincronização cardíaca.

#### Desfechos econômicos secundários

Os desfechos econômicos secundários incluem: i) comparação de cada componente de custo listado na Tabela 2 entre os grupos de tratamento, a fim de identificar os principais determinantes da variação no custo médico direto total; e ii) uma análise de impacto orçamentário estimando o efeito anual no orçamento de saúde do Brasil se todos os pacientes elegíveis para TRC recebessem ESC em vez de EBV.

#### Desfecho econômico exploratório e estimativas de qualidade de vida

As análises exploratórias examinarão associações entre características basais importantes e o custo médico direto total, visando identificar preditores de maior custo. Em conformidade com as diretrizes de custo-efetividade,^29 , 30^ os *quality-adjusted life-years* (QALYs) serão derivados das avaliações do questionário EuroQol 5-Dimension aplicadas no início, aos 6 meses e aos 12 meses, com utilidades calculadas com base em um conjunto de valores da população brasileira.^31 - 33^

#### Tamanho da amostra

O tamanho da amostra-alvo para o desfecho clínico primário do ensaio foi de 180 participantes; o recrutamento foi concluído após 179 randomizações em dezembro de 2023. Nenhum cálculo separado de tamanho de amostra foi realizado para o desfecho econômico, uma vez que este foi designado como desfecho secundário.

#### Coleta de dados

O uso de recursos será medido utilizando uma estrutura de microcusteio, com métodos distintos para a hospitalização inicial e para o período de seguimento. Para a hospitalização inicial, será aplicado o método de custeio baseado em atividades e tempo ( *time-driven activity-based costing* , TDABC) para quantificar o consumo de recursos e estimar custos unitários, complementado pela extração de dados de faturamento hospitalar. Durante o seguimento, o uso de recursos será registrado sempre que um participante for hospitalizado ou necessitar de atendimento urgente por descompensação da ICFER, utilizando um formulário eletrônico de relato de caso ( *eCRF* ) no REDCap™.^34 , 35^

A Tabela 2 resume todos os recursos e insumos de custo incluídos na análise. Procedimentos detalhados para o TDABC e para a contabilidade baseada em recursos consumidos são descritos a seguir.

#### Time-driven activity-based costing para TRC

O TDABC é uma abordagem de mensuração de custos que enfatiza a utilização de mão de obra e de instalações, com o objetivo de aprimorar a precisão das estimativas de custo e a compreensão do fluxo de cuidado. O método será implementado na seguinte sequência: i) mapear o fluxo de cuidado e suas principais atividades; ii) identificar todos os recursos consumidos nesse fluxo; iii) estimar o custo total de cada recurso; iv) para mão de obra e uso de instalações, determinar a capacidade horária e calcular a taxa de custo unitário (TCU; $/h; por exemplo, custo com ocupação de leito por hora); v) estimar o tempo de uso de cada recurso ao longo do fluxo; vi) calcular o custo total por paciente; e vii) agregar os custos para o conjunto do estudo. Essa sequência, juntamente com a etapa inicial de identificação da tecnologia avaliada, segue um modelo validado de oito etapas para o TDABC.^20^

**Figura 1:**
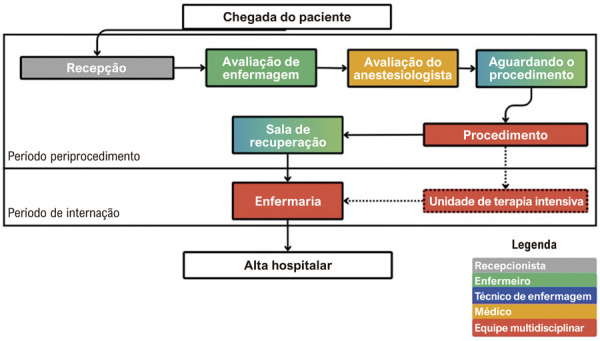
– Atividades do paciente durante a hospitalização inicial. Cada caixa representa um conjunto de recursos de utilização de mão de obra e de instalações empregados ao longo do fluxo de cuidado. As cores e legendas indicam as categorias de profissionais de saúde envolvidas em cada etapa. As linhas pontilhadas que levam à Unidade de Terapia Intensiva representam etapas opcionais que ocorrem apenas em alguns casos. Durante as fases de Espera pelo Procedimento e Sala de Recuperação, tanto enfermeiros quanto técnicos de enfermagem participam do cuidado ao paciente, conforme representado pelas cores azul e verde mescladas.

A Figura 1 apresenta o mapa preliminar do fluxo de cuidado. Uma equipe multidisciplinar de eletrofisiologistas, cardiologistas e pesquisadores de avaliação de tecnologias em saúde validará o mapa; em seguida, cada centro participante o revisará para considerar diferenças locais de prática clínica que possam influenciar custos específicos.

Para cada instalação hospitalar do fluxo, as TCUs serão derivadas com apoio dos departamentos financeiros, compilando custos de mão de obra e uso de instalações. Os custos de pessoal serão baseados nos salários dos profissionais envolvidos no atendimento; quando necessário, serão utilizadas fontes secundárias, como relatórios salariais públicos (em hospitais públicos) e editais recentes de contratação. Os custos de uso de instalações (excluindo pessoal) refletirão o número de leitos e as horas de funcionamento em que os departamentos prestam assistência. Será assumida uma capacidade ociosa de 10%. Dados de faturamento hospitalar serão extraídos para quantificar os custos de insumos, medicamentos e exames (laboratoriais e não laboratoriais) durante o período perioperatório, excluindo margens de lucro e custos de dispositivos.

Na etapa 5, o tempo gasto pelos pacientes em cada área hospitalar antes, durante e após o procedimento será registrado no eCRF do procedimento índice dentro do REDCap™. Em todos os centros, um investigador local acompanhará cada paciente durante o procedimento e coletará, dos prontuários, os horários de entrada e saída em cada unidade (listadas na Figura 1 ). Conforme práticas de avaliações econômicas baseadas em ensaios clínicos e orientações especializadas,^20 , 36 - 40^ o tempo de mão de obra profissional consumida por paciente será estimado como função do tempo de permanência em cada área hospitalar, aplicando padrões de cuidado específicos para cada unidade, com base em observações diretas em casos selecionados e entrevistas com gestores hospitalares (um exemplo hipotético é apresentado no Apêndice Suplementar ).

Por fim, para cada recurso, a TCU será multiplicada pelo tempo registrado de uso, somando-se esses valores aos custos de insumos, medicamentos e exames extraídos dos registros hospitalares para obter o custo por paciente.

#### Método adicional de contabilidade baseada em recursos

Os custos dos dispositivos foram determinados no início do estudo em acordo com a BIOTRONIK, refletindo valores de mercado vigentes; para análise, serão ajustados para refletir os preços de mercado sob a perspectiva do pagador no Brasil (público e privado). Se a estratégia de TRC inicialmente designada falhar e for necessário cruzamento, serão contabilizados custos adicionais de fornecimento com o dispositivo.

Nos cruzamentos precoces (durante o procedimento ou a internação inicial), os custos hospitalares aumentados serão capturados via TDABC. Nos cruzamentos tardios (após a alta), os custos da hospitalização inicial serão replicados, ajustando os custos dos dispositivos para refletir o novo procedimento de TRC.

Durante o seguimento, visitas de emergência e hospitalizações por descompensação da ICFER serão registradas, e os custos estimados conforme a Classificação Brasileira Hierarquizada de Procedimentos Médicos (CBHPM). A CBHPM, desenvolvida pela Associação Médica Brasileira e apoiada por diversas sociedades profissionais, incluindo o Conselho Federal de Medicina, fornece um parâmetro de referência para alinhar valores de reembolso aos custos institucionais reais nos setores público e privado.^41 , 42^

Os custos do manejo ambulatorial da ICFER serão obtidos de estudos prévios de microcusteio detalhado com TDABC e padronizados conforme a classe funcional da NYHA na linha de base e aos 6 meses.^43^

## Análise estatística

Os custos dos dispositivos e outras variáveis de custo por centro (p.ex., TCU das instalações) serão consolidados em um banco de dados por paciente. O custo médico direto total por paciente será calculado conforme especificado na Equação S1.^20^

Os dados ausentes serão tratados por meio de uma estratégia hierárquica adaptada à importância da variável e ao mecanismo de ausência.^15 , 44 - 46^ As variáveis principais com potencial impacto sobre o desfecho econômico primário incluem custos dos dispositivos, variáveis de tempo de uso das instalações hospitalares (principal indicador de consumo de recursos e mão de obra profissional durante as atividades hospitalares) e custos associados aos desfechos de interesse (reinternações e visitas urgentes por ICFER).

Para variáveis de tempo de instalação, será tolerado até 10% de dados ausentes; caso esse limite seja excedido, será aplicada imputação múltipla, assumindo ausência ao acas.^44 - 46^ Por desenho, não se esperam dados ausentes para custos de dispositivos nem para custos relacionados aos desfechos de interesse. Para outras variáveis de custo com mais de 10% de ausência, será utilizada imputação pela mediana, dado seu impacto limitado sobre o custo total e o custo médio por grupo, também assumindo ausência ao acaso.^44 - 46^

## Minimização de custos versus razão custo-efetividade incremental

Dado que a hipótese principal do estudo é de não inferioridade para os desfechos clínicos (ESC vs. EBV), a análise primária será de minimização de custos. Se surgir diferença estatisticamente significativa nos desfechos clínicos, será conduzida uma análise exploratória de custo-utilidade (ou dominância), estimando a razão custo-efetividade incremental em QALYs (ESC vs. EBV). Essa análise adotará uma perspectiva de tempo de vida, baseada em modelo, utilizando os resultados do estudo como insumos primários.^29 , 30^

Na análise principal de minimização de custos, as diferenças nas médias de custo médico direto total entre os grupos serão avaliadas por meio de um modelo linear generalizado com ligação logarítmica e distribuição gama, acompanhado de *bootstrap* não paramétrico (1.000 reamostragens) para derivar intervalos de confiança (ICs) de 95%, reconhecendo a assimetria esperada com caudas longas.^15 , 23 , 47 , 48^ As especificações do modelo poderão ser adaptadas com base nas distribuições observadas ao final do seguimento. Serão relatados os custos médios com ICs 95% e as medianas (intervalos interquartis) por grupo.

Para os desfechos econômicos secundários, serão relatados os custos médios por recurso ( Tabela 2 ) com ICs 95% via *bootstrap* , além das medianas (intervalos interquartis). A análise de impacto orçamentário, estimando o gasto anual se todos os pacientes elegíveis para TRC no Brasil recebessem ESC em vez de EBV, será conduzida sob a perspectiva do sistema de saúde brasileiro, integrando os custos do estudo com estimativas epidemiológicas da população elegível.

Modelos exploratórios avaliarão preditores de custo total, adicionando características clínicas e sociais relevantes, incluindo determinantes sociais de saúde e comorbidades, ao modelo linear generalizado; os resultados serão apresentados como riscos relativos com ICs 95%.^15 , 23 , 47 , 48^

## Potenciais análises de sensibilidade

Análises adicionais poderão variar os preços dos dispositivos de acordo com perspectivas de compra pública vs. privada e reestimarão custos para os desfechos de interesse utilizando a Tabela SIGTAP do SUS para procedimentos, medicamentos e outros insumos, em conformidade com estudos anteriores.^49^

## Ética e disseminação

Um comitê independente de monitoramento de dados e segurança, apoiado por uma equipe estatística imparcial, tem acesso aos dados não cegos e fornece supervisão contínua. O Comitê Executivo é responsável pela apresentação dos resultados e pela redação do manuscrito. O estudo foi aprovado por comitês de ética em pesquisa institucionais. Em conformidade com a Declaração de Helsinque da Associação Médica Mundial, todos os participantes forneceram consentimento livre e esclarecido por escrito.

## Discussão

A mensuração precisa de custos é essencial para análises econômicas e para o planejamento de alocação de recursos. Ela apoia o reembolso adequado e esclarece o consumo de recursos ao longo de fluxos específicos de cuidado, permitindo identificar oportunidades de redesenhar serviços de acordo com as necessidades dos pacientes e custos sustentáveis.^50^ Essa necessidade torna-se ainda mais urgente diante do aumento dos gastos em saúde nas Américas, especialmente com doenças cardiovasculares.^51 - 53^

Na América Latina, prevê-se que os gastos em saúde aumentem 2,8 vezes até 2050, impulsionados principalmente por condições cardiovasculares, e já têm crescido mais rapidamente que o produto interno bruto em vários países;^51 , 53^ na América do Norte, estima-se que os gastos com doenças cardiovasculares quadripliquem até 2050.^52^ Essas tendências representam desafios significativos para PBMR na América Latina, onde o esforço para alcançar cobertura universal de saúde, e acesso equitativo a tecnologias avançadas, pressiona ainda mais a sustentabilidade financeira.^51 , 53 , 54^

As consequências são relevantes para indivíduos com condições crônicas de alta gravidade que requerem terapias de custo elevado, como a ICFER, levando a maiores gastos diretos do próprio bolso nos PBMR. Na América Latina, esses pagamentos representam cerca de 40% dos gastos totais em saúde (31% no Brasil), em comparação com 11% nos Estados Unidos e 14% no Canad.^53 , 55^ Essa pressão financeira contribui para a não adesão e o sub-uso de terapias eficazes (p.ex., TRC) – mesmo sendo considerada custo-efetiva em cenários de PBMR–, resultando em piores desfechos.^14 , 56 - 58^

Por exemplo, um ano após a alta hospitalar por ICFER no Brasil, apenas cerca de 40% dos pacientes com ICFER estavam recebendo medicamentos essenciais.^56 , 59^ Além disso, a taxa anual de procedimentos de TRC por milhão de habitantes no SUS permanece abaixo da faixa esperada de 100-200 por milhão de pessoas.^10 , 11^ Essas observações reforçam a necessidade de análises precisas de custos e estratégias de economia que otimizem a alocação de recursos, ampliem o acesso no cenário cardiovascular das Américas e possibilitem tratar mais pacientes.

Embora valiosa, a coleta de dados de custo precisos em ensaios clínicos é desafiadora, especialmente quando há ausência de dados individuais em variáveis essenciais para a estimativa de custos (p.ex., tempo de permanência em instalações hospitalares e consumo de insumos por paciente).^20 , 36 , 37^ Nosso estudo busca mitigar esses desafios. Este protocolo metodológico oferece um modelo claro, incorporado ao ensaio clínico, para a obtenção de dados de custo de mão de obra, infraestrutura e insumos, juntamente com variáveis de tempo em instalações registradas diretamente no eCRF.

Uma estratégia hierárquica para lidar com dados ausentes foi especificada, e o plano estatístico para quantificação de incerteza, testes de hipóteses e predição segue as melhores práticas atuais para análise de dados de microcusteio em ensaios clínicos.

A aplicação do TDABC em um ensaio multicêntrico, em que os desfechos econômicos têm papel central, pode facilitar a adoção mais ampla de métodos robustos de mensuração de custos e promover maior equidade no acesso à TRC entre pacientes com ICFER. De forma mais ampla, a mensuração precisa de custos em ensaios clínicos pode subsidiar iniciativas baseadas em valor e programas correlatos, fortalecendo a tomada de decisão e melhorando a acessibilidade ao cuidado cardiovascular.^17 , 20 , 22 , 36 , 60^

## Conclusão

O estudo PhysioSync-HF, que compara a ESC e a EBV, foi projetado para identificar oportunidades de redução de custos capazes de ampliar o acesso equitativo à TRC para pacientes com ICFER. O protocolo também oferece uma estrutura reprodutível para avaliações econômicas baseadas em ensaios clínicos, aplicável a diferentes condições cardiovasculares.

## SUPPLEMENTARY APPENDIX

*Material suplementar Para informação adicional, por favor, clique aqui. https://abccardiol.org/supplementary-material/2025/12212/2025_supplement.pdf
