# Supplementary material for: Estimulação do Sistema de Condução Versus Estimulação Biventricular na Insuficiência Cardíaca Crônica: Protocolo para a Análise Econômica do Estudo PhysioSync-HF
Source: Arq Bras Cardiol. 2026 Jan 26;122(12):e20250254. [Article in Portuguese] doi: 10.36660/abc.20250254 (PMC12978376; doi:10.36660/abc.20250254)
Supplement: *Material suplementar [file 0066-782x-abc-122-12-e20250254-suppl01.pdf]

SUPPLEMENTARY APPENDIX.

Conduction system pacing vs. biventricular resynchronization in chronic heart failure: Protocol for the economic analysis of the PhysioSync-HF trial

Economic protocol of the PhysioSync-HF trial

Figure S1. Brazilian map with participating cities.

Equation S1. The mathematical equation represents the total direct medical cost per patient.

Supplemental Methods S1. Case example of time variables measurement.

Figure S1. Brazilian map with participating cities.

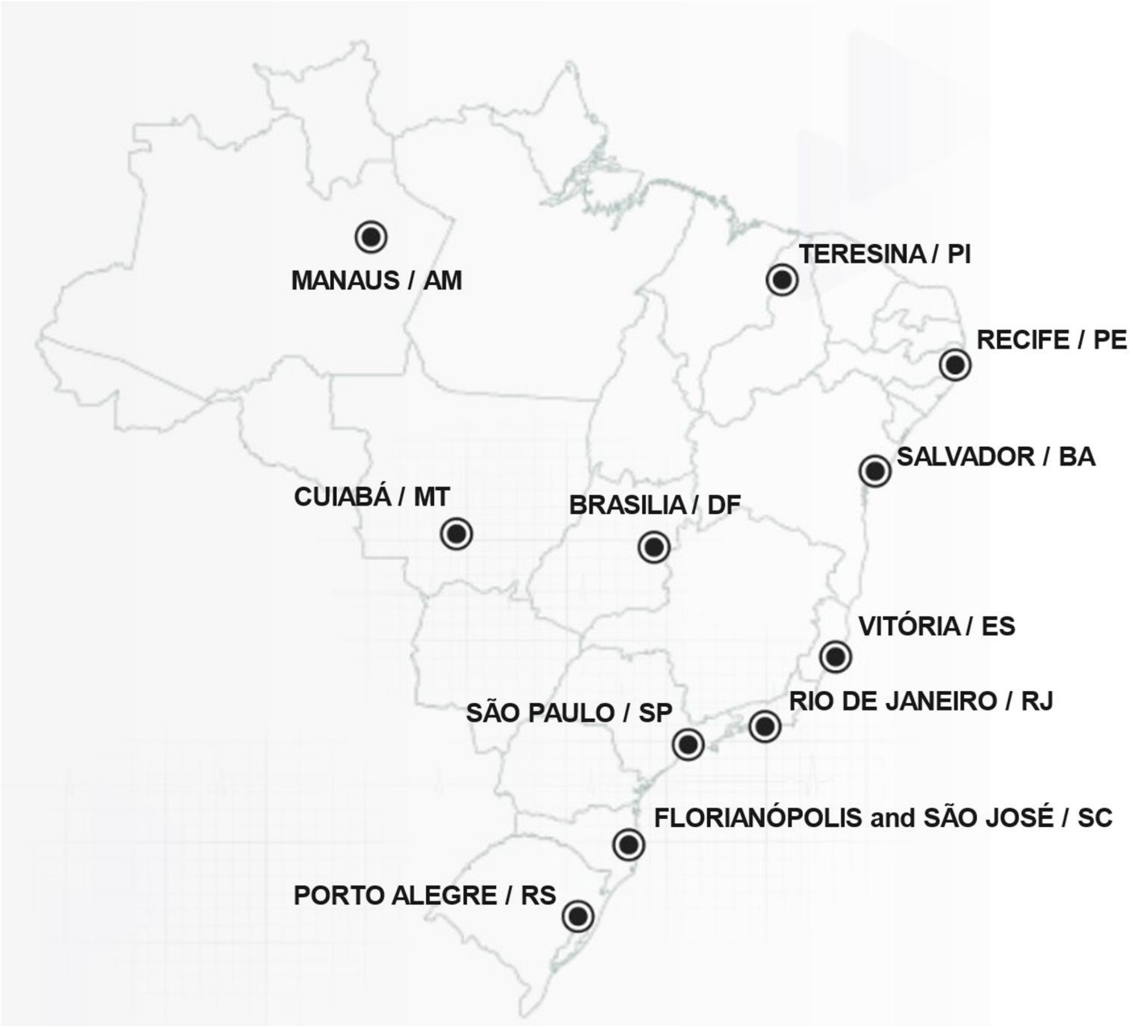

Acronyms of Brazilian states are described as follows: AM - Amazonas; BA - Bahia; DF - Distrito Federal; ES - Espírito Santo; MT - Mato Grosso; PE - Pernambuco; PI - Piauí; RJ - Rio de Janeiro; RS - Rio Grande do Sul; SC - Santa Catarina; SP - São Paulo.

Equation S1. The mathematical equation represents the total direct medical cost per patient.

$$\text{Total Cost} = \sum T_i \times CCR_i + y + d + c + o + Ha + Hv =$$

$$\text{Total Cost} = \sum T_l \times CCR_l + \sum T_f^{***} \times CCR_f + y + d^{***} + c + o + Ha^{***} + Hv^{***} =$$

Legend. *T:* time of resource utilization; *CCR*: Cost Capacity Rate per resource ($/hour); *i*: number of resources; *l*: number of labor resources; *f*: number of facility utilization resources; *y*: direct costs associated with the intervention (i.e., medications, exams, other supplies); *d:* device costs; *c*: additional costs with cross-over; *o:* costs of heart failure management in outpatient care; *Ha*: mean estimate of costs with hospitalizations due to decompensated heart failure; *Hv*: mean estimate of costs with urgent visits due to decompensated heart failure; ***key variables.

Supplemental Methods S1. Case example of time variables measurement.

Patient X received the trial intervention and their time variables for facility utilization (T_f, Eq. 1), according to our care flow map, were a pre-procedure period fixed time of 70 minutes, a 120 minutes procedure period ($T_{f(procedure)}$), 120 minutes in the recovery room, and 48 hours in the hospital ward after the procedure ($T_{f(hospital\ ward)}$). Using the measurement of the nurse labor time consumption ($T_{l(nurse)}$, Eq. 1) as an illustration, a hypothetical estimate would be the following: in the default routine of care, the nurse will spend 20 minutes per hour in the pre-procedure phase, will participate in the procedure, will visit the patient 1 time in the recovery room (20 minutes) per hour, and will visit the patient 3 times, 20 minutes each, per day in the hospital ward. Thus, the final $T_{l(nurse)}$ cost driver would be: 20 minutes + $T_{f(procedure)}$ + 20 + (3 x 20 x $T_{f(hospital\ ward))}$) = 260 minutes, assuming facility utilization procedure time equal to nurse procedure time (available during the all period), and 48 hours in a hospital ward.

This case exemplifies how labor time variables will be fulfilled as a function of facility utilization times, assuming default care routines (which will be defined by direct observation and interviews with participant centers). We believe that this last assumption is reasonable to ensure data completeness, without detriment to the final economic outcome, increasing feasibility for our trial-based multicenter economic evaluation.
