# Supplementary material for: Estimulação do Sistema de Condução Versus Estimulação Biventricular na Insuficiência Cardíaca Crônica: Protocolo para a Análise Econômica do Estudo PhysioSync-HF
Source: Arq Bras Cardiol. 2026 Jan 26;122(12):e20250254. [Article in Portuguese] doi: 10.36660/abc.20250254 (PMC12978376; doi:10.36660/abc.20250254)
Supplement: Translation

# Translation: Conduction System Pacing vs Biventricular Pacing in Chronic Heart Failure: Protocol for the Economic Analysis of the PhysioSync-HF Trial

**DOI:** 10.36660/abc.20250254i

**Authors:** Sérgio R. R. Decker, Ana Paula Beck da S Etges, André Zimerman, Fernanda D. Alves, Caique M. Ternes, Juliana S. Santos, Leandro Zimerman, Luis Eduardo Rohde, Alexander Dal Forno, André d’Avila, Dhruv S. Kazi, Eduardo G. Bertoldi, Carisi A. Polanczyk

**Affiliations:** 1 Brazil Serviço de Clínica Médica do Hospital Moinhos de Vento, Porto Alegre, RS – Brazil; 2 USA Smith Center for Outcomes Research, Beth Israel Deaconess Medical Center and Harvard Medical School, Boston – USA; 3 Brazil Programa de Pós-Graduação em Cardiologia e Ciências Cardiovasculares, Universidade Federal do Rio Grande do Sul, Porto Alegre, RS – Brazil; 4 Brazil MOVE Academic Research Organization, Hospital Moinhos de Vento, Porto Alegre, RS – Brazil; 5 Brazil Programa de Pós-Graduação em Epidemiologia, Universidade Federal do Rio Grande do Sul, Porto Alegre, RS – Brazil; 6 Brazil Projetos de Pesquisa, Hospital Moinhos de Vento, Porto Alegre, RS – Brazil; 7 Brazil Serviço de Cardiologia, Hospital Moinhos de Vento, Porto Alegre, RS – Brazil; 8 Brazil Hospital SOS Cardio, Florianópolis, SC – Brazil; 9 USA Cardiac Arrhythmia Service, Beth Israel Deaconess Medical Center and The Harvard Thorndike Electrophysiology Institute, Boston – USA; 10 Brazil Faculdade de Medicina, Universidade Federal de Pelotas, Pelotas, RS – Brazil; 11 Brazil Hospital de Clínicas de Porto Alegre, Porto Alegre, RS – Brazil; 12 Brazil Instituto para Avaliação e Translação em Saúde para doenças Crônicas e negligenciadas de Alta Relevância, IATS-CARE, Porto Alegre, RS – Brazil

**Keywords:** Cardiac Resynchronization Therapy, Systolic Heart Failure, Health Care Costs

## Abstract

**Background:**

Conduction system pacing (CSP) has emerged as an alternative to biventricular pacing (BVP) for cardiac resynchronization therapy (CRT), with potential clinical benefits and lower costs. PhysioSync-HF is a multicenter, randomized trial comparing these strategies from both clinical and economic perspectives in patients with heart failure with reduced ejection fraction (HFrEF).

**Objective:**

To describe the rationale and design of the trial-based economic evaluation embedded within the PhysioSync-HF trial.

**Methods:**

The PhysioSync-HF trial enrolled 179 patients with 1-year follow-up. Procedural cost data will be collected using a time-driven activity-based costing approach. Costs associated with the device, adverse clinical events, and ambulatory care during follow-up will be estimated using resource-based accounting methods. Appropriate methods will address missing data, and statistical analyses will account for the skewed distribution of cost variables.

**Results:**

The primary economic outcome is the between-group difference in total direct medical costs per patient over the 1-year follow-up (CSP vs BVP). Secondary outcomes include component-level cost breakdowns of direct medical expenses and a budget impact analysis estimating the annual effect on Brazil’s health care system if all eligible patients received CSP instead of BVP.

**Conclusion:**

By leveraging a multicenter cardiovascular trial to measure costs of CSP versus BVP, this economic evaluation aims to identify cost-saving opportunities that could expand equitable access to CRT for individuals with HFrEF in Brazil, while providing insights relevant to other health care settings worldwide.

**Trial Registration:**

NCT05572736.

## Introduction

Heart failure with reduced ejection fraction (HFrEF) is a chronic condition affecting about 1.3% of the global population across all ages, with higher estimates of 3.4% among adults and 8.3% among older adults.^1^Owing to population aging, the number of affected individuals continues to rise.^2^Currently, about 600 per 100,000 people live with HFrEF in Southern Latin America, with nearly double that prevalence in North America, contributing to a substantial and growing clinical and economic burden.^1 - 4^

Within HFrEF, patients with conduction abnormalities represent a higher-risk subgroup for whom individualized therapies confer important benefits.^5^Cardiac resynchronization therapy (CRT) reduces morbidity and mortality in patients with HFrEF and left bundle branch block (LBBB).^5 - 8^Biventricular pacing (BVP) is the standard technique for delivering synchronized pacing, whereas conduction system pacing (CSP) — which directly stimulates the intrinsic conduction system — has recently emerged as a potential alternative.^9^

From an economic standpoint, CSP may reduce procedural costs by requiring fewer supplies. This consideration is particularly relevant in low- and middle-income countries (LMICs), such as Brazil, where an estimated 200,000 individuals are eligible for CRT but only a small fraction receive it.^3 , 10 , 11^High procedural costs and complexity have limited global CRT implementation and contribute to inequality in access among people living with HFrEF.^12 - 14^

Embedding economic data collection within clinical trials can align the adoption of new technologies by regulatory bodies with their societal value.^15 - 17^Given the expanding clinical and economic burden driven by demographic change and the rise of noncommunicable diseases — led by cardiovascular diseases^3 , 18 , 19^— this approach is particularly important because it enables more precise cost quantification through micro-costing methods and can catalyze broader improvements in health-care management.^15 , 16 , 20 - 22^

However, trial-based cost assessments face challenges, including heterogeneous data-collection and analytic practices, as well as the limited feasibility of detailed measurements which can lead to high rates of missing data.^23 - 26^These epidemiologic and methodological issues underscore the need for clear methodological descriptions and protocol publications for trial-based economic evaluations.

This manuscript aims to describe the economic evaluation embedded in the PhysioSync-HF trial (NCT05572736), which compares CSP with BVP in patients with HFrEF. Our central hypothesis is that CSP will be cost-saving relative to BVP from a health-system perspective and may reduce cost-related barriers to CRT referral and access.

## Methods

### Study design

PhysioSync-HF is a multicenter, randomized, noninferiority trial comparing CSP with BVP in patients with HFrEF and LBBB. A key secondary endpoint is the between-arm difference in total direct medical costs per patient, assessed from the perspective of the Brazilian health system ( Table 1 ), encompassing both the public sector (Brazilian Unified Health System, SUS) and the private sector.

**Table 1:** – Perspective of cost assessment including total direct medical costs

Cost component	Included elements	Perspective	
		Health care	Societal
Direct health-care costs paid by third-party payers	Drugs/devices; hospital services; physician services; exams	x	x
Direct health-care costs paid out-of-pocket by patients	Drugs/devices; hospital services; physician services; exams	x	x
Direct non-medical costs	Patient time; unpaid caregiver time; transportation		x
Indirect costs	Losses of productivity; cost of premature mortality		x
Other indirect or intangible costs	Legal; environmental; other		x

We highlight the costs evaluated in this trial-based economic analysis, which are categorized as direct medical expenses.

Costs include those related to the CRT procedure and subsequent follow-up care. Central Illustration presents the trial schema and data-collection plan for the embedded economic evaluation. Methods are reported in accordance with the Standard Protocol Items: Recommendations for Interventional Trials and, where relevant to economic analyses, elements of the Consolidated Health Economic Evaluation Reporting Standards.^27 , 28^The full trial protocol has been registered with ClinicalTrials.gov (NCT05572736).

A total of 179 patients were enrolled between November 2022 and December 2023. Economic data collection and analyses will cover the 12-month follow-up. Principal investigators, study coordinators, and research directors will remain blinded to economic results until database lock. The trial is supported by the Brazilian Ministry of Health through the Support Program for Institutional Development of the Unified Health System (Proadi-SUS).

### Study setting and participant centers

Enrollment occurred in both inpatient and outpatient settings across 14 sites in 11 Brazilian states (Figure S1). All participating centers received standardized training for economic data collection.

### Study population

Eligible participants are adults (≥ 18 years) with HFrEF, left ventricular ejection fraction (LVEF) ≤ 35%, New York Heart Association (NYHA) functional class II-III symptoms, and LBBB with QRS duration ≥ 130 ms. All participants must be on optimized guideline-directed medical therapy and have a referral for CRT.

### Study intervention

Participants were randomized 1:1 to CSP or BVP. The CSP strategy includes LBBB area pacing, His-bundle pacing, or deep septal pacing; if electrical resynchronization remained incomplete, left bundle branch-optimized CRT (LOT-CRT) with an additional coronary-sinus lead was permitted. BVP programming followed contemporary guideline-based practice and was performed by the electrophysiology team. Crossover was allowed if the assigned strategy proved unsuccessful.

### Outcomes

#### Clinical outcomes ‘

The primary clinical outcome of PhysioSync-HF is a hierarchical composite of all-cause mortality, heart-failure hospitalization, urgent heart-failure visit, and change in LVEF from baseline to 12 months. Noninferiority of CSP versus BVP will be tested first, using a margin defined as an odds ratio < 1.20. If noninferiority is demonstrated, superiority will be tested sequentially for the same outcome.

#### Primary economic outcome

The primary economic outcome (key secondary trial endpoint) is the difference in total direct medical costs per patient between treatment arms. Total direct medical costs comprise the components listed in Table 2 . Analyses will use a modified intention-to-treat population at 12 months, including all randomized patients who underwent the index procedure, thereby capturing costs related to early or late crossovers after randomization.

**Table 2:** – Resources and cost estimates measured during the PhysioSync-HF trial

Resource	Source of cost estimate (base case)	Potential sensitivity analysis
Index hospitalization		
CRT device	Wholesale Acquisition Cost	Public vs private purchasing perspective
Labor utilization^*^	TDABC	—
Facility utilization^†^	TDABC	—
Medications	Hospital claims	—
Exams	Hospital claims	—
Other supplies	Hospital claims	—
Follow-up after discharge		
HF outpatient care	Ghisleni et al. ^36^	—
Urgent visits for decompensated HF	CBHPM ^45^	SIGTAP ^35^
Hospitalization for decompensated HF	CBHPM ^45^	SIGTAP ^35^

*Labor utilization includes physician and non-physician labor costs. †Facility utilization includes anesthesia-recovery room, electrophysiology laboratory, ward, and intensive care unit costs. CBHPM: Brazilian Hierarchical Classification for Medical Procedures (health-cost benchmark); CRT: cardiac resynchronization therapy; HF: heart failure; TDABC: time-driven activity-based costing; SIGTAP: Unified Health System’s Table for Procedures, Drugs, and Other Supplies (public-sector benchmark).

#### Secondary economic outcomes

Secondary economic outcomes include the following: i) comparison of each cost component in Table 2 by treatment arm to identify drivers of variation in total direct medical costs; and ii) a budget-impact analysis estimating the annual effect on Brazil’s health care budget if all CRT-eligible patients received CSP rather than BVP.

#### Exploratory economic outcome and quality-of-life estimates

Exploratory analyses will examine associations between key baseline characteristics and total direct medical costs to identify predictors of higher cost. Consistent with cost-effectiveness guidelines,^29 , 30^quality-adjusted life-years (QALYs) will be derived from EuroQol 5-Dimension Questionnaire assessments administered at baseline, 6 months, and 12 months, with utilities calculated using a Brazilian population value set.^31 - 33^

#### Sample size

The target sample size for the trial’s primary clinical endpoint was 180 participants; enrollment concluded after 179 randomizations in December 2023. No separate sample-size calculation was performed for the economic endpoint because of its designation as a secondary outcome.

#### Data collection

Resource use will be measured using a micro-costing framework with distinct methods for the index hospitalization and the follow-up period. For the index hospitalization, time-driven activity-based costing (TDABC) will be applied to quantify resource consumption and estimate unit costs, supplemented by hospital claims extraction. During follow-up, resource utilization will be recorded whenever a participant is hospitalized or requires an urgent visit for decompensated HFrEF using an electronic case report form (eCRF) in REDCap™.^34 , 35^

Table 2 summarizes all resources and cost inputs included in the analysis. Detailed procedures for TDABC and resource-based accounting are provided below.

#### TDABC for the CRT

TDABC is a cost-measurement approach that emphasizes labor and facility utilization to improve the accuracy of cost estimates and the understanding of the care pathway. We will implement TDABC in the following sequence: i) map the care pathway and its main activities; ii) identify all resources consumed along this pathway; iii) estimate the total cost of each resource; iv) for labor and facility use, determine hourly capacity and calculate the capacity cost rate (CCR; $/h; eg, cost related to bed occupancy per hour); v) estimate the time each resource is used along the pathway; vi) compute total cost per patient; and vii) aggregate costs for the trial cohort. This sequence — together with the initial step of identifying the technology under assessment — aligns with a validated eight-step framework for TDABC.^20^

Figure 1 shows the preliminary care-flow map. A multidisciplinary team of electrophysiologists, cardiologists, and health technology assessment researchers will first validate this map; each participating center will then review it to account for site-level differences in default practices that may influence local costs.

**Figure 1:** – Patient activities during the index hospitalization. Each box represents a set of labor and facility utilization resources used throughout the care pathway. Colors and labels indicate the categories of health professionals involved at each stage. The dotted lines leading to the Intensive Care Unit represent optional steps that occur only in some cases. During the Waiting for Procedure and Recovery Room stages, both nurses and nurse technicians participate in patient care, as represented by the blended blue and green colors.

For each hospital facility in the pathway, CCRs will be derived with support from the centers’ financial departments by compiling labor and facility-use costs. Labor costs will be based on the salaries of professionals involved in patient care; when needed, secondary sources will include publicly available salary reports (for public hospitals) and recent recruitment notices. Facility-use costs (excluding personnel) will reflect the number of beds and the operating hours during which departments provide patient care. We will assume 10% idle capacity.

Hospital claims and billing data will be extracted to quantify resources and costs for supplies, drugs, and tests (laboratory and non-laboratory) during the perioperative period, excluding profit margins and device costs.

For step 5, the time patients spend in each hospital activity before, during, and after the procedure will be captured in the index-procedure eCRF within REDCap™. At all centers, a local investigator will follow each patient through the procedure and abstract entry and exit times from medical records for each facility (i.e., hospital facilities the patient undergo during activities, listed in Figure 1 ). Consistent with prior trial-based economic evaluations and expert guidance,^20 , 36 - 40^we will estimate labor time per patient as a function of the time spent in each hospital facility, applying standards of care for each facility based on direct observations in a subset of cases and interviews with hospital leaders (a hypothetical example is provided in the Supplementary Appendix ).

Finally, for each resource we will multiply the CCR by the recorded time of use and then sum these amounts with the costs of supplies, medications, and exams from hospital claims to obtain the per-patient cost.

#### Additional resource-based accounting method

Device costs were determined at trial initiation in agreement with BIOTRONIK, reflecting current market values; for analysis, they will be adjusted to reflect the wholesale acquisition cost from the payer perspective in Brazil (public and private). If the initially assigned CRT strategy fails and a crossover is required, we will account for additional device-supply costs.

For early crossovers (during the index procedure or index hospitalization), elevated hospitalization costs will be captured via TDABC. For late crossovers (post-discharge), we will replicate index-hospitalization costs and adjust device costs to reflect the subsequent CRT procedure.

During follow-up, urgent emergency-department visits and hospitalizations for decompensated HFrEF will be recorded, and costs will be estimated using the Brazilian Hierarchical Classification for Medical Procedures (CBHPM). CBHPM, developed by the Brazilian Medical Association and supported by multiple professional societies including the Federal Council of Medicine, provides a benchmark to align reimbursement fees with actual institutional costs in both public and private sectors.^41 , 42^

Costs associated with outpatient heart failure management will be sourced from prior bottom-up micro-costing studies using TDABC and standardized by NYHA class at baseline and 6 months.^43^

## Statistical analysis

Device costs and other site-level cost variables (eg, facility CCRs) will be consolidated into a patient-level dataset. Total direct medical cost per patient will be calculated as specified in Equation S1.^20^

Missing data will be handled using a hierarchical strategy tailored to the variable importance and missingness mechanisms.^15 , 44 - 46^Key variables with potential impact on the primary economic outcome include device costs, facility-time variables (the main proxy for resource and labor time consumed during hospital activities, and costs associated with outcomes of interest (heart-failure rehospitalization and urgent visits).

For facility-time variables, up to 10% missingness will be tolerated; if exceeded, multiple imputation will be employed, assuming missing at random.^44 - 46^By design, we do not anticipate missing data for device costs or for costs linked to outcomes of interest. For other cost variables with > 10% missingness, median imputation will be used, given their expected limited influence on total costs and mean costs per arm, again assuming missing at random.^44 - 46^

## Cost minimization vs incremental cost-effectiveness ratio

Since the trial’s noninferiority hypothesis for clinical outcomes (CSP vs BVP), the primary analysis is a cost-minimization comparison. If a statistically significant difference in clinical outcomes emerges, we will conduct an exploratory cost-utility (or dominance) analysis, estimating the incremental cost-effectiveness ratio for QALYs (CSP vs BVP). This analysis will adopt a lifetime perspective using a model-based approach, with trial results as primary inputs.^29 , 30^

For the main cost-minimization analysis, differences in mean total direct medical costs between arms will be assessed using a generalized linear model with log link and gamma family, accompanied by nonparametric bootstrap (1,000 resamples) to derive 95% CIs, acknowledging the expected right-skew with heavy tails.^15 , 23 , 47 , 48^Model specifications may be adapted based on observed distributions at the end of follow-up. We will report mean costs with 95% CIs and median (interquartile range) by arm.

For secondary economic outcomes, we will report mean cost per resource ( Table 2 ) with 95% CIs via bootstrap as well as median (interquartile range). The budget-impact analysis — estimating annual spending if all Brazilian CRT-eligible patients received CSP vs BVP — will be conducted from the Brazilian health-system perspective by integrating trial-based costs with epidemiologic estimates of the eligible population.

Exploratory models may evaluate predictors of total cost by adding key patient characteristics, including social determinants of health and comorbidities, to the generalized linear model; results will be presented as relative risks with 95% CIs.^15 , 23 , 47 , 48^

## Potential sensitivity analyses

Additional analyses may vary device prices for public vs private purchasing perspectives and re-estimate costs for outcomes of interest using the SUS’s Table for Procedures, Drugs, and Other Supplies (SIGTAP), consistent with prior work.^49^

## Ethics and dissemination

An independent data and safety monitoring board, supported by an impartial statistical team, has access to unblinded data and provides continuous oversight. The Executive Committee is responsible for presenting results and preparing the manuscript. The trial received approval from institutional review boards. In accordance with the World Medical Association Declaration of Helsinki, all participants provided written informed consent.

## Discussion

Accurate cost measurement is essential for economic analyses and resource allocation. It supports appropriate reimbursement and clarifies resource consumption along specific care pathways, enabling identification of opportunities to redesign services around patients’ needs at sustainable costs.^50^This imperative is heightened by rising health-care spending across the Americas, particularly for cardiovascular disease.^51 - 53^

In Latin America, health spending is projected to increase 2.8-fold by 2050, driven largely by cardiovascular conditions, and has risen faster than gross domestic product in several countries;^51 , 53^in North America, spending on cardiovascular conditions is projected to quadruple by 2050.^52^These trends pose substantial challenges for LMICs in Latin America, where the push for universal health coverage and equitable access to advanced technologies further strains affordability.^51 , 53 , 54^

The consequences are tangible for individuals with chronic, high-severity conditions that require costly therapies, such as HFrEF, leading to higher out-of-pocket expenditures in LMICs. In Latin America, out-of-pocket payments account for about 40% of total health spending (31% in Brazil), compared with 11% in the United States and 14% in Canada.^53 , 55^These financial pressures contribute to nonadherence and underuse of effective therapies such as CRT — even though CRT is considered cost-effective in LMIC settings–, with consequent worse outcomes.^14 , 56 - 58^

For example, 1 year after discharge for heart-failure hospitalization in Brazil, only about 40% of patients with HFrEF were receiving pivotal medications,^56 , 59^and the annual rate of CRT procedures per million population in the SUS remains below the expected indication range of about 100-200 per million people.^10 , 11^These observations underscore the need for precise cost analyses and cost-saving strategies to optimize resource allocation, expand access across the American cardiovascular landscape, and potentially treat more patients.

Although valuable, collecting accurate cost data in clinical trials is challenging, particularly when addressing patient-level missingness in variables required for cost estimation (eg, time spent in each activity during the index hospitalization for CRT, and per-patient supply consumption).^20 , 36 , 37^Our study seeks to mitigate these challenges. This methodological protocol provides a clear, trial-embedded template for acquiring labor, facility, and supply cost data, together with time variables captured directly within the eCRF.

A hierarchical strategy for handling missingness is specified, and the statistical plan for uncertainty quantification and hypothesis testing, and prediction follows current best practices for analyzing micro-costing data in trials.

Implementing TDABC within a multicenter trial — where economic outcomes are central — may facilitate broader adoption of robust costing methods and promote equity in access to CRT among patients with HFrEF. More broadly, accurate costing within trials can inform value-based initiatives and related programs, strengthening decision-making and improving the affordability of cardiovascular care.^17 , 20 , 22 , 36 , 60^

## Conclusion

The PhysioSync-HF trial comparing CSP with BVP is designed to identify cost-saving opportunities that can enhance equitable access to CRT for patients with HFrEF. The protocol also offers a reproducible framework for trial-based economic evaluations that can be applied across cardiovascular conditions.

## SUPPLEMENTARY APPENDIX

*Material suplementar Para informação adicional, por favor, clique aqui. https://abccardiol.org/supplementary-material/2025/12212/2025_supplement.pdf
